# Five weeks of Yuishinkai karate training improves balance and neuromuscular function in older adults: a preliminary study

**DOI:** 10.1186/s13102-022-00458-6

**Published:** 2022-04-11

**Authors:** Hajer Mustafa, Aimee Harrison, Yao Sun, Gregory E. P. Pearcey, Bruno Follmer, Benjamin M. Nazaroff, Ryan E. Rhodes, E. Paul Zehr

**Affiliations:** 1 Rehabilitation Neuroscience Laboratory, University of Victoria, Room 172, McKinnon Building, 3800 Finnerty Road, Victoria, BC V8P 5C2 Canada; 2 School of Exercise Science, Physical and Health Education, University of Victoria, Victoria, BC Canada; 3 Human Discovery Science, International Collaboration on Repair Discoveries (ICORD), Vancouver, BC Canada; 4 Behavioural Medicine Laboratory, University of Victoria, Victoria, BC Canada; 5 Centre for Biomedical Research, University of Victoria, Victoria, BC Canada; 6 Division of Medical Sciences, University of Victoria, Victoria, BC Canada

**Keywords:** Posture, Balance, Hoffmann reflex, Spinal cord excitability, Martial arts, Rehabilitation, Aging

## Abstract

**Background:**

Martial arts training has shown positive impacts on balance and physiological measurements. Further investigation of the contents and feasibility of an effective therapeutic assessment of martial arts is needed in older adults, mainly for future applications and real-world implementation.

**Methods:**

Sixteen older adults (8 male, 8 female, age 59–90 years), with or without chronic conditions, participated in a preliminary study using 5-weeks of karate training and a triple baseline control procedure. Group and single subject data analyses were conducted for dynamic balance, Timed Up and Go (TUG), hand grip, ankle plantarflexion force, and spinal cord excitability (via the soleus H-reflex) pre- and post-training.

**Results:**

On average, participants completed a total of 2437 steps, 1762 turns, 3585 stance changes, 2047 punches, 2757 blocks, and 1253 strikes. Karate training improved dynamic balance performance such that the group average time was reduced (time to target (−13.6%, *p* = 0.020) and time to center (−8.3%, *p* = 0.010)). TUG was unchanged when considering the entire group (*p* = 0.779), but six participants displayed significant changes. Left handgrip (7.9%, *p* = 0.037), and plantarflexion force in the right (28.8%, *p* = 0.045) and left leg (13.3%, *p* = 0.024) increased for the group. Spinal cord excitability remained unchanged in group data analysis but 5 individuals had modulated H_max_/M_max_ ratios.

**Conclusion:**

5-weeks of karate training delivered in a fashion to mimic generally accessible community-level programs improved balance and strength in older adults. Whole-body movement embodied in karate training enhanced neuromuscular function and postural control. We met the overriding goal of this preliminary study to emphasize and assess feasibility and safety for the generalizability of martial arts interventions to real-world communities to impact health outcomes. Further quantitative work should explore threshold dose and development of martial arts training interventions as potential “exercise is medicine” functional fitness for older adults.

## Background

Falls are the second leading cause of accidental deaths worldwide and are the largest comorbidity in people ages 65 years or older [1, 2]. Dynamic postural control is critical for quality of life, yet aging is associated with alteration of neuromuscular function and reduced functional capacity [2–4]. As a consequence, many therapeutic programs focus on enhancing balance, functional movements, and strength training to improve postural control and reduce the risk of falls [5–7]. Additional options for accessible, community-based training programs to maintain or enhance integrated body function across the lifespan and after impairment are needed [1]. With this in mind, it is of utmost importance that older adults, with or without chronic conditions, are able to access training that will lead to longevity and long-term therapeutic benefits. In this context, we define therapeutic to mean physical training that produces a benefit to mitigate against the loss of functional capacity (such as related to postural control) occurring across the lifespan and exacerbated in chronic health conditions (such as Parkinson’s disease or stroke). Within the context of “exercise is medicine” several reviews have emphasized the benefits of exercise therapy for individuals with differing conditions with the general goal of creating individualised training focused on balance, gait, strength, and functional ability training [8].

Martial arts are typically thought of as recreational exercise or self-defense training. However, practice usually involves training in bodyweight shifting, stepping, changing stance and other movements that challenge balance. The sequences of choreographed martial arts movements, called kata, or forms, are a key component of martial arts training and can be practiced alone without any equipment. Since this type of practice can be easily administrated in a community setting, many studies have investigated the effects of martial arts on balance and other physiological aspects of health. Within the literature, there have been many studies with Tai Chi Chuan in older adults but the research with karate is increasing. Exposure to Shotokan karate training over 8-weeks with older adults and individuals with Parkinson’s Disease (PD) improved strength, cognitive wellness, and static balance of older adults [9, 10]. These studies evaluated demographic data, cognitive, and emotional variables through assessments of psychological measures and balance motor function through a one-leg stand [10]. Following training, there were improvements in static balance for the PD participants and some small changes in emotional and cognitive aspects [9, 10]. Physiological improvements were also observed after Tai Chi training in older adults and individuals with chronic conditions, such as PD [11–15]. However, the effects of karate on neurophysiology, dynamic balance reactions, and clinical aspects of health require further documentation [9, 10].

To be useful as potential exercise therapy emphasis needs to continue to be shifted to the health benefits martial arts training provides. Further information is also required on the generalizability of various martial arts training approaches that are commonly found in communities around the world. A limitation to the general application of martial arts as exercise therapy are lack of details about minimum exercise stimulus and the corresponding timelines and feasibility of application in a general heterogenous population as seen in the community. For example, detailed accounting of numbers of repetitions of martial arts techniques and the duration of training are critical for replication and extension. In work where martial arts have been used as the training stimulus and showed positive impacts on participants’ balance and psychological measurements, explicit details about what the participants practiced are sparse or absent [9, 10]. Investigation of effective therapeutic stimulus of martial arts is needed with documentation of movement tracking to advance the knowledge for future applications and real-world implementation. Additionally, the duration of training interventions has varied considerably from 5 weeks to 3 years [5–7, 9–11, 16–28] and there is no consistent record of different training protocols such as the intensity, rest intervals, and applications of techniques. For further application, better documentation of activities undertaken, assessment of minimum timing for efficacious outcome, and extension to more commonly found community martial arts (e.g. hard-style systems like karate, hapkido, Shaolin kung fu systems) are needed.

The purpose of this preliminary study was to assess feasibility and safety for the generalizability of martial arts interventions to real-world communities to impact health outcomes. Additionally, we sought to assess the effects of short term, documented karate training on balance performance and lower limb musculoskeletal function in older adults with or without chronic conditions. We focused on the health application of the martial arts training rather than direct skill acquisition in karate. We predicted that the requirements of postural control in karate training would strengthen neurophysiological integrity and have beneficial effects on dynamic balance reactions and movement in older adults.

## Methods

### Participants

Sixteen older adults (8 male, 8 female, age 59–90 years; 171.2 ± 4.7 cm; 68.1 ± 8.9 kg), with or without chronic conditions, participated in this preliminary study and each informed written consent under a protocol approved by the University of Victoria Human Research Ethics Board (Protocol #18-213). An additional intake was cancelled due to the COVID 19 pandemic. There were five older adults with chronic conditions (4 male, 1 female; 3 Parkinson’s disease, 1 vascular dementia, 1 stroke). Participants were recruited via posters, email, in-person events, and word of mouth. Inclusion criteria were that participants did not use a pacemaker and could stand independently without assistance. Exclusion criteria were previous or ongoing mind–body (e.g. any form of martial arts, qigong, or yoga) exercise experience. Participants completed the following assessments pre and post training in the subsequent order: spinal cord excitability, hand grip strength, plantarflexion force, dynamic balance, and Timed Up and Go (TUG) for integrated function.

### Study design and control procedures

**Fig. 1:**
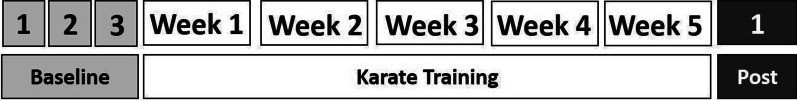
Illustration of the assessment and karate intervention training protocol. A multiple baseline within-subject control design was used

The testing and the training spanned nine weeks for each participant (Fig. 1). Baseline tests to establish control measures were performed in the first three weeks (one test per week) in the Rehabilitation Neuroscience Laboratory at the University of Victoria, followed by five weeks of karate training (one-hour sessions on Monday, Wednesday, and Friday yielding 15 sessions total) in the dance studio at the University of Victoria. The post-training tests took place on week 9. To ensure each participant received sufficient attention and guidance from the instructors and volunteers, participants were trained as two cohorts, therefore the whole study lasted six months in total. The karate sessions were delivered by black-belt instructors experienced in teaching martial arts in community-based settings. This schedule (5 weeks of training, 15 sessions total) was used to replicate both the approach taken in our previous strength and locomotor training interventions conducted [29–34] as well as to mimic the timing and delivery taken in other community-based martial arts programs [9, 17, 26, 28]. Since therapeutic applications often necessitate time efficiency, preference for a short duration for compliance and demonstration of efficacy was chosen.

A multiple baseline approach, as well as a within-participant control design, was performed as in recent studies [29–31, 35–37]. The three pre-training assessments allow participants to create a baseline of their variability that enables them to act as their own control. This reduces the impact of between-subject variability and allows for single participant statistical analysis using 95% confidence intervals from the pre-training data. The triple baseline procedure provides higher internal consistency of measures, decreased variability compared to using a control group and also provides measures of individual variability that can be used for single-subject analysis [29, 30, 34, 37]. The order of test administration, time of the day, and other environmental conditions were consistent for each participant and testing sessions. In addition, participants were asked not to start any new forms of exercise throughout the training, but they were also asked to maintain their pre-existing exercise routines.

### Karate training contents

Each karate training session consisted of a warm-up involving general stretches and movements of the arms and legs followed by practicing individual techniques of punches, step punching, open hand striking, and blocking. Instruction and explanation of the fighting applications of all techniques were also provided to create additional context for the movements practiced. The bulk of the practice was repetition (~ 45–60 s per cycle) of the kata “Pinan Nidan”. This kata consists of 13 stepping, 11 turning, 7 punching, 2 striking and 13 blocking movements in 9 directions and with 21 stance changes. The version used is from the traditional Japanese Yushinkai system founded in 1948 by Grandmaster Gansho (Motokatsu) Inoue (1918–1993), but many karate styles include this kata and related patterns in their training curriculum. Pinan Nidan was selected because it emphasizes whole-body movement, bodyweight shifting and stance changes, but does not include any static single leg standing or kicking. Across many styles and systems, this kata is commonly introduced first to new karate learners. For our participants, this kata provides sufficient challenge on their standing balance with minimal risk of falling. The movements and choreography were done at a slower speed and modified to ensure that the participants were following along. By the end of the second week, all the participants had learned the full kata and performing repetitions at a normal speed.

During each session, an average of 10 repetitions of Pinan Nidan was completed with a minimum of 150 kata performances across the 5-week training period. Movements were done at slow speed and modified according to the range of motion, functional ability, and neurological status of each participant. Ten volunteer “spotters” were distributed amongst the participants to provide support when needed, usually in a 1:1 ratio or sometimes 1:2 if a volunteer was absent. The repetitions of each technique (e.g. step, block, punch, etc.) practiced by the participants were documented by two researchers. Documentation of the training sessions was done with written record of each type of movement performed for each participant, while video footage was also obtained to confirm any uncertainties. The training contents varied daily so it was a goal to record all motions for all the participants and quantify what was done over the 5 weeks.

### Balance assessment

A commercially available balance board (Wii Balance Board, Nintendo, Kyoto, Japan) was used with customized software (LabVIEW 2011 National Instruments, Austin, TX, USA), and data were sampled at 100 Hz. The validity (r = 0.99) and reliability (ICC = 0.88) of balance board has been confirmed [38] and used in several previous studies to assess postural control [36, 39, 40].

During the dynamic balance assessment, each participant stood barefoot on the balance board, feet at shoulder width, eyes open, and hands on the hips. Center of pressure was displayed on a laptop screen as a white dot that was in front of the participants. The trial began with a target (red) dot on the screen and moved in a random sequence amongst eight cardinal and ordinal directions. Participants were instructed to shift their weight distribution so that their center of pressure met the target as quickly and accurately as possible. Following the initial practice trial, participants completed five trials with one to five minutes of rest between the intervals. The time to reach the target (tTarget), time to return to center from target (tCenter), and the sum of tTarget and tCenter (tTotal) were obtained and the average of the five trials was used for analysis. Detail description of balance assessment methods can be found in previous work [39–41].

### Clinical assessment

The TUG test was used as a clinical measure of functional capacity. TUG is a simple test that investigates the mobility of an individual through the evaluation of both static and dynamic balance [42]. The test is known to assess the fall risk and measure the progress of balance, sit to stand, and walking in elderly individuals, primarily those with neurological or chronic conditions [42]. For this test, participants were sitting in a chair with their arms on the armrests. There was a line on the floor 3 m away. When the experimenter said “GO” the participants stood up out of the chair, walked to the target then made their way back. The timer began when we told them to begin walking and ended once they had done the loop and sat back into the chair.

### Strength measures

Grip strength was assessed using commercial handgrip dynamometers (Right: Takei Scientific Instruments Company Ltd., Niigata, Japan; Left: Lafayette Instrument Co.). Participants were in a seated posture with one arm relaxed on the lap and the test arm extended with palm facing downward and shoulder abduction at a 45° angle. Measurements were alternated between the right then left hand to avoid fatigue with three trials completed on each side. During each trial, the participant performed maximal isometric contraction for 5 s. Strength at the ankle was assessed through maximal voluntary isometric contractions of ankle plantarflexion force using strain gauge load cells (Omegadyne Ltd., Model 101-500) while the participant was seated in a custom-fit chair with foot straps to minimize movement and the ankle at 90° [30, 43]. Muscle activation from the soleus, tibialis anterior, and vastus lateralis muscles were recorded with a customized LabVIEW program (National Instruments, Austin, TX, USA).

### Muscle activity and spinal cord excitability

Hoffmann (H-) reflexes were evoked as proxy measures of spinal cord excitability while participants stood with both feet flat on the floor. The reflexes were measured from a standing position to be task-specific to the training position [44]. An overhead harness system was utilized to ensure that there was no risk of falling. Electromyography was collected using bipolar surface electrodes placed bilaterally on the tibialis anterior, vastus lateralis, and soleus muscles while a ground electrode was placed over the right or left patella. Recordings were amplified (500 or 1000 times for soleus and 5000 times for other muscles) and filtered (10–1000 Hz for soleus and 100–300 Hz for others) (P511 Grass Instruments, AstroMedInc, West Warwick, RI, USA) and sampled at 2.5 kHz on a computer running customized software (LabVIEW, National Instruments, Austin, TX, USA).

The tibial nerve on the dominant leg was stimulated at the popliteal fossae using 1 ms square wave pulses to evoke H-reflexes in the soleus. Bipolar surface electrodes were used for stimulation delivered pseudo randomly 3–5 s apart for all trials using a Digitimer (Mendtel, NSW, Australia) constant current stimulator (model DS7A). A non-contact milliammeter (mA-2000, Bell Technologies, Orlando, FL, USA) was used to measure the current delivered for each stimulus. A recruitment curve was collected by continuously increasing stimulation intensity until at least three maximal M-waves were recorded. Participants monitored the electromyography level on a computer at 10% of the maximal voluntary contraction for plantarflexion which was determined before the experiment. M-wave and H-reflex (M-H) recruitment curve was obtained from 40 stimulations. The maximal peak-to-peak H-reflex amplitude (H_max_) and M-wave (M_max_) were determined [31]. Overall reflex excitability was determined by calculating the H_max_/M_max_ ratio. Mmax values used in this ratio were calculated by taking the mean of the three largest M-waves from the recruitment curve. If the 3rd largest M-wave amplitude was not within 10% of the amplitude of the largest M-wave, a mean of the 2 largest M-waves was used. Hmax was calculated as the single largest peak to peak amplitude of H-reflex from the recruitment curve.

### Data analysis and statistical methods

All statistical analyses were performed using SPSS (v.24, Armonk, NY: IBM Corp.). All the participants were analysed as one group, then single subject analyses were done for all participants and categorized based on if they were neurologically intact or if they had a chronic condition. For group comparisons, the three baseline sessions were first compared via one-way Repeated Measures Analysis of Variance (rmANOVA). We tested the assumption of sphericity via Mauchly’s Test (*p* > 0.05) and if violated, degrees of freedom and p-value were corrected using the Greenhouse–Geisser method. When there were no differences between the three baseline tests, an averaged baseline value was calculated and compared to the post-training results through paired t-test, as done in our previous training studies [29–31]*.* The difference between post and the mean of baseline values were expressed as percent change from the averaged baseline results (%∆). For single subject analysis and group comparisons, a 95% confidence interval (CI) was established from the triple baseline, as done in our previous clinical [31] and other training intervention [29–32] studies. The 95% CI approach is also endorsed for single-subject analysis [45–47]. Post-training values were then compared to the respective 95% CI and considered statistically significant if they fell outside this range. Effect size (Cohen’s *d*) was calculated to provide a standardized magnitude of changes [48]. Small (0.2–0.5), medium (> 0.5–0.8), and large (> 0.8) effect sizes descriptors were used as in a similar study [17]. Results are presented with overall group data first followed by descriptions of the outcomes of the single subject analysis [31]. The level of significance was set at *p* < 0.05, with group data reported to 3 decimal places and *p* < 0.05 for the single subject and the group 95% CI analyses.

## Results

### Training repetitions completed

**Fig. 2:**
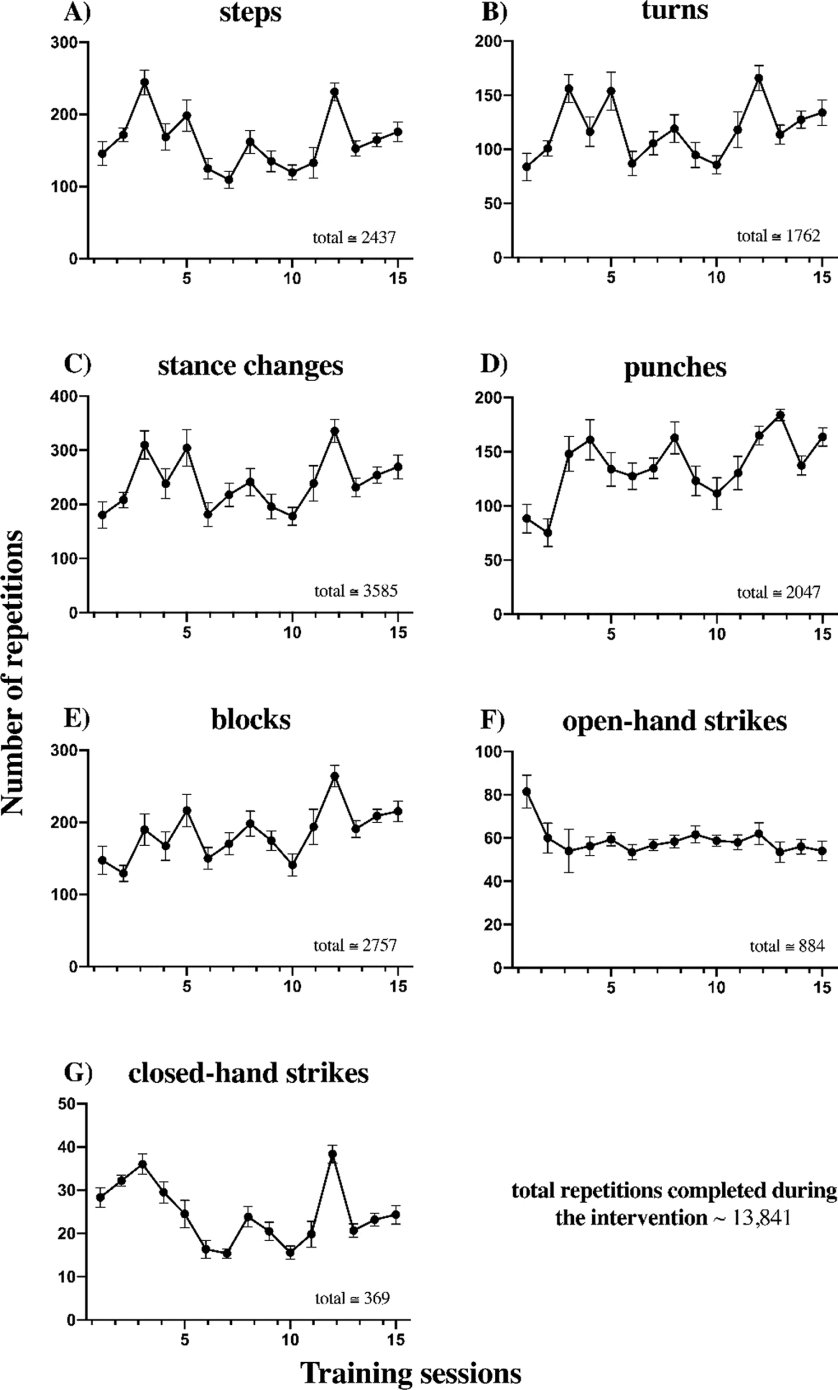
Daily movement repetitions completed during warm-up and Pinan Nidan practice. Movements were categorized into different karate techniques. Daily group averages are displayed in the figures and the total number of repetitions completed throughout the training is presented in the bottom right of each panel. Each panel represents the following: **a** steps (including those to do techniques like punches or blocks), **b** turns (e.g. to the side, to the back), **c** stance changes (e.g. from front stance to cat stance), **d** punches (on the spot or stepping), **e** blocks (to high, middle, and low levels), **f** open-hand strikes (e.g. sword hand), and **g** closed-hand strikes (e.g. hammerfist)

The participants had a total average of 2437 steps, 1762 turns, 3585 stance changes, 2047 punches, 2757 blocks, 884 open-hand strikes and 369 closed-hand strikes, with a total of 13,841 movements throughout the intervention Fig. 2. No adverse events caused by the training paradigm was observed or reported by the participants or their caregivers.

### Dynamic balance assessment

**Fig. 3:**
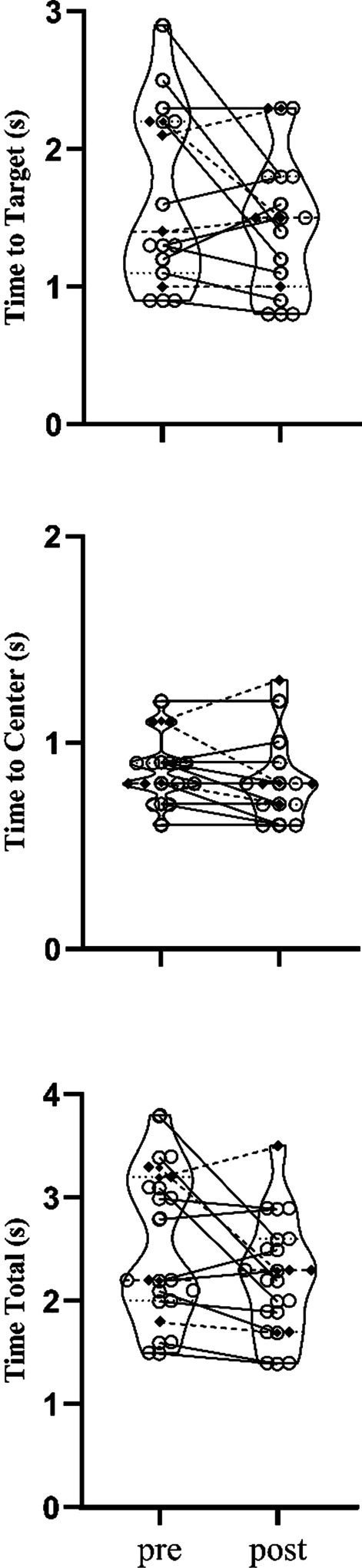
Time to reach the target (tTarget), to get back to center (tCenter) and the sum of them (tTotal) in the dynamic postural control test. The open circles represent the neurologically intact older adults, while the filled diamonds and dotted lines represent the older adults with chronic conditions

Training generally improved dynamic balance performance. The group average time for dynamic balance reaction reduced by -13.6% for tTarget (t(14) = 1.742; *p* = 0.020; *d* = 0.450), −8.3% for tCenter (t(14) = 2.959; *p* = 0.010; *d* = 0.763), and −11.8% for tTotal (t(14) = 2.071; NS *p* = 0.057; *d* = 0.535), respectively (Fig. 3). The 95% CI analysis of the group data showed that there were significant changes for tTarget, tCenter, and tTotal. Pre-training ANOVA showed a difference between the baseline tests for tTarget (F_2,28_ = 4.518, *p* = 0.020), tCenter (F_2,28_ = 6.8, *p* = 0.004) and tTotal (F_2,28_ = 5.601, *p* = 0.009).

One neurologically intact older adult participant had significant dynamic balance reaction reduction for tTarget (−44.4%, *p* < 0.05), three for tCenter (−15.9%, −20.6%, −14.4%; *p* < 0.05) and two for tTotal (−34.9%, −35.9%; *p* < 0.05). For the older adults with PD, two participants showed significant reductions for tCenter (−11.5% and −26.4%; *p* < 0.05) and one for tTarget (−33.7%; *p* < 0.05), neither showed individual changes in tTotal. The participant with vascular dementia had −5.3% (*p* > 0.05) reduction for tCenter and increased in time for tTarget by 11% (*p* > 0.05) and tTotal 4.9% (*p* > 0.05). The chronic stroke participant showed significant increase for tCenter (12%; *p* < 0.05) and no individual changes in tTarget or tTotal.

### Clinical assessment

Group data for the TUG were unchanged following the intervention (t(15) = −0.369; *p* = 0.717; *d* = 0.092) and the 95% CI did not show changes either (0.6%, *p* > 0.05). Four of the neurologically intact older adults showed significant reduction in time for TUG (−6.3%, −7.8%, −4.5%, −8.6%; *p* < 0.05), while seven had no change (7.2%, 1%, 5.4%, −2.2%, 0.8%, 4.0%; *p* > 0.05). One of the PD participants showed significant increased time in the TUG test (3.8%, *p* < 0.05). The individual with vascular dementia had no change and the chronic stroke participant had a significant increase in TUG (14%, *p* < 0.05). Pre training control was achieved since ANOVA showed no differences between the triple baseline for the TUG (F_2,30_ = 0.252, *p* = 0.779).

### Strength measures

**Fig. 4:**
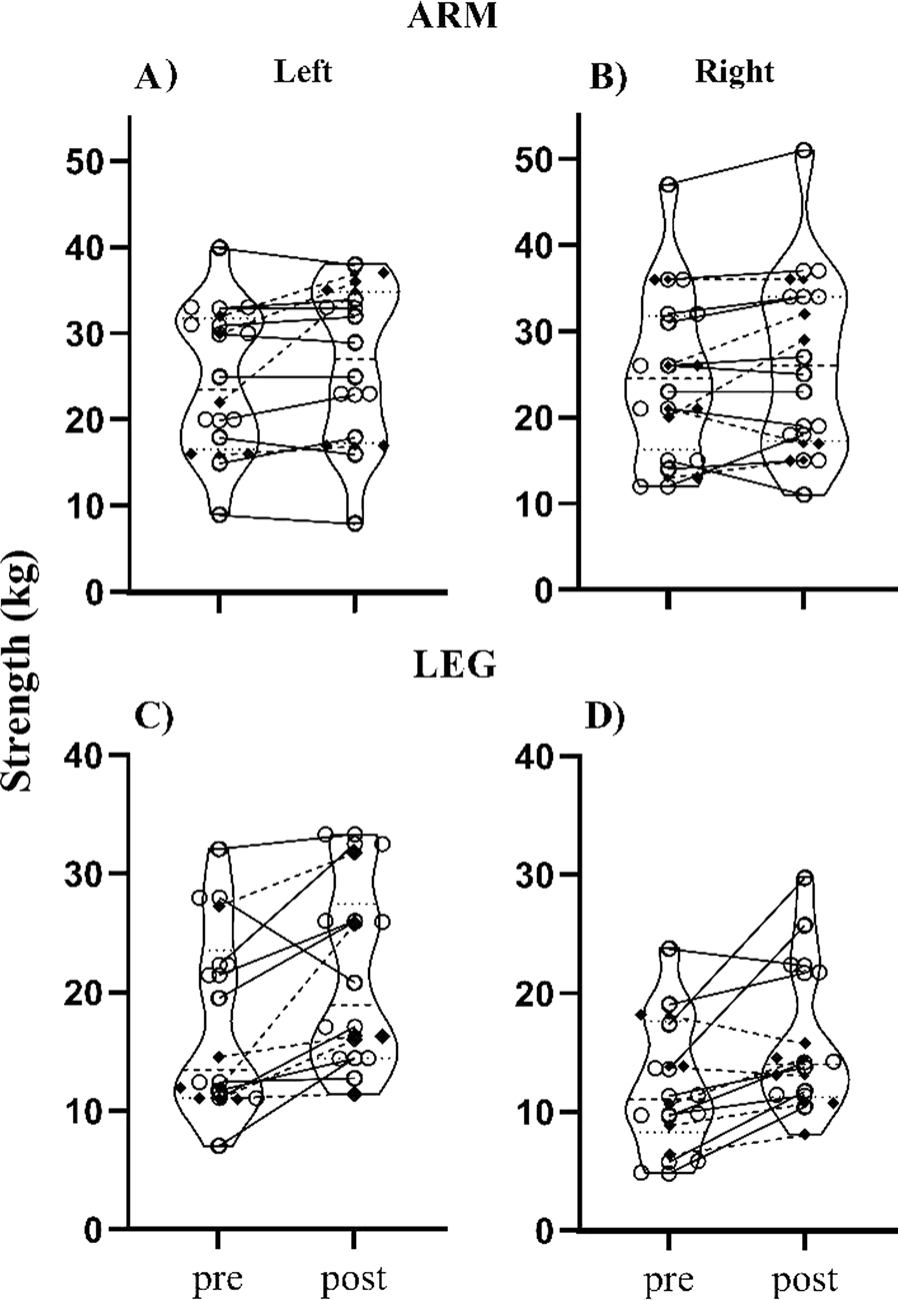
Pre-post comparison of strength, measures force in kilogram (kg), in the arms and legs. Each panel represents the following: **a** Left Arm, **b** Right Arm, **c** Left Leg, and **d** Right Leg. Open circles = Neurologically Intact Older Adults; Filled Diamonds = Older Adults with Chronic Conditions

In the group, ankle plantarflexion strength significantly increased on both right leg (28.8%; t(10) = −2.286; *p* = 0.045; *d* = 0.689) and left leg (13.3%; (t(11) = −2.616; *p* = 0.024; *d* = 0.755), respectively (Fig. 4, panels C and D). Evaluation of the group data from the 95% CI showed that there were significant differences for the right and left legs (*p* < 0.05). Across the group, smaller changes were found for the left arm (7.9%; t(15) = −2.260; *p* = 0.039; *d* = 0.563) and in the right arm (6.3%; t(15) = −1.765; *p* = 0.098; d = 0.440), with the 95% CI analyses showing changes for the left and right arms as well (Fig. 4, panels A and B). Baseline control was achieved since ANOVA showed no differences between the baseline measures for the right arm (F_2,30_ = 0.479, *p* = 0.624), left arm (F_2,30_ = 0.067, *p* = 0.935), right leg (F_2,20_ = 0.210, *p* = 0.812), and left leg (F_2,22_ = 0.183, *p* = 0.834).

Five neurologically intact participants had significant increases in the right leg (88.7%, 70.7%, 114.2%, 101.6%, 28.8%; *p* < 0.05) and five had significant increases in the left leg strength (45.8%, 54.1%, 21.3%, 104.8%, 32.9%; *p* < 0.05). Significant improvement of arm strength was observed in five neurologically intact older adults in the right arm (10.0%, 52.8%, 10.1%, 5.1%, 4.8%; *p* < 0.05) and three in the left arm (20.5%, 17.3%, 4.1%; *p* < 0.05).

Two participants with PD showed significant increases in right leg force (36.3%, 26.1%; *p* < 0.05) and all three had significant increases in left leg force (115.8%, 12.2%, 45.9%; *p* < 0.05). Two participants with PD showed significant increases (42.6%, 19.0%; *p* < 0.05) and one with a significant decrease (−17.9%, *p* < 0.05) in right arm strength. One PD participant had significantly increased strength for the left arm (18.9%, *p* < 0.05).

The individual with vascular dementia showed significant (*p* < 0.05) increases for the right leg (20.8%), right arm (20.8%), and left arm (57.4%), respectively. The chronic stroke participant had a significant increase for left arm strength (15.1%; *p* < 0.05).

### Spinal cord excitability

Gross assessment of spinal cord excitability, as observed by the H_max_/M_max_ ratios, were unchanged across the group. The pre-training averages for H_max_/M_max_ ratios were 15% while the post-training was 18.6% (∆23.7%; (t(12) = −1; *p* = 0.335; *d* = 0.279), and the group post data was outside of the 95% CI established at baseline. Pre training control was achieved since the ANOVA test showed no differences between the triple baseline (F_2,26_ = 0.665, *p* = 0.523).

**Fig. 5:**
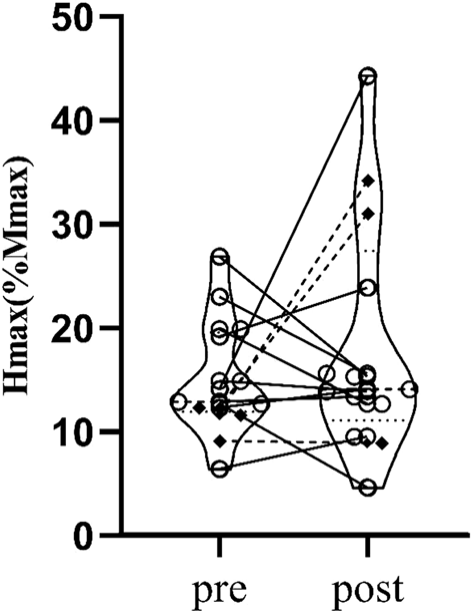
Pre-post comparison of spinal cord excitability. Open circles = Neurologically Intact Older Adults; Filled Diamonds = Older Adults with Chronic Conditions

Two of the neurologically intact older adults showed significant increases in spinal cord excitability (215.4%, 47.9%; *p* < 0.05), while one showed a significant decrease (−35.9%; *p* < 0.05). Two PD participants had significant increases in spinal cord excitability (152.4%, 195.3%; *p* < 0.05). No individual changes were observed in the vascular dementia and chronic stroke participants (Fig. 5).

## Discussion

A realistic community-level of martial arts training program appears feasible and safe and thus suggests the generalizability of martial arts interventions to real-world communities to impact health outcomes. Our limited data set suggests martial arts as part of a therapeutic application for improving balance in older adults with and without chronic conditions requires and is worthy of further refinement and assessment in future studies.

### Dynamic balance and postural corrections improved by karate training

Following the training, most of the participants showed improved performance in postural control through a decrease in time taken for dynamic postural reaction during the balance test. This enhanced dynamic balance performance leads to better postural control in older adults with or without chronic conditions, which is congruent with similar studies that observed the stabilizing effects of martial arts [4, 17, 20, 21, 42]. Balance requires complex neuromechanical integration of muscular, somatosensory, visual, and vestibular coordination [3, 21, 49–51]. The dynamic balance data suggest that this multisensory integration could be enhanced or improved by karate training but further testing of visual and somatosensory components is needed. The Pinan Nidan kata﻿ focuses on shifting of body weight distribution and transition between different stances, so the maintaining and strengthening of balance control were anticipated. Studies of martial arts such as Tae Kwon Do and Ving Tsun (Wing Chun) improved hand grip strength, balance, and gait in older adults of similar ages to those in the current study [20, 21, 52].

Postural control is key for the prevention of falls in elderly populations; therefore, the changes are correlated with improved skeletal and proprioceptive muscle strength that minimizes the risk of injuries [3, 50–53]. Coordination of different limbs is needed for the enhancement of gait control, which can be accelerated through exercises involving whole-body movements [3, 11, 20, 51, 54, 55]. As the data suggest, quicker time for the dynamic balance test infers that participants may have gained more stability in their legs which could minimize the risk of falls [21, 25]. Additionally, improvements in balance correlate to the musculoskeletal control in various muscles in the body [21], suggesting that there was faster motor neuron recruitment and increased motor neuron excitability.

Karate kata requires both upper and lower limbs to move in a coordinated pattern, this type of movement may enhance the neural coupling between the oscillators in each limb that subserving rhythmic movements [55, 56]. Our previous works indicated that movements that utilizing the interlimb neural connection, such as arm or arm-leg cycling can amplify the neural plasticity in people with chronic stroke [29, 30, 33]. It has also been confirmed that the excitability in the spinal pathways is reduced with aging with smaller H_max_ and M_max_ amplitudes observed in older adults [57]. Here, we did not observe any change in the H-reflex amplitude from the group data, however, significant increases were observed in 5 participants including 2 participants with PD. We speculated that the coordinated upper and lower limbs movements can potentially amplify the spinal circuit in older adults.

### Effects on muscle strength

Strength was improved in both the arms and the legs, which aligns with previous research that showed rehabilitation with arm and leg cycling improved walking function after training [30, 31]. Strength in the arms and legs augmented after the 5-week training protocol, and we speculate that influences from descending and interlimb pathways may have been amplified due to exercise. Prior work with chronic stroke participants evaluated how the linkages between central pattern generator networks required for arm and leg movement during locomotion improved with arm and leg cycling training [29, 30]. It is conceivable that such central pattern generator networks and related interlimb connections might be enhanced by the karate training [29, 30, 58].

The improved strength in both the arms and legs of the participants highlights that whole-body movement without the use of machinery can allow for changes in neuromuscular performance. The locomotor circuits evoke the supraspinal and spinal regulatory mechanisms that are needed for activities such as walking [30, 34, 44]. The primary goal of rehabilitation following neurotrauma is to regain function in movements such as walking and it is also important to strengthen these connections with aging to avoid the risk of falls [1]. Training through holistic approaches involving whole-body movement could maintain or enhance the intrinsic systems that exist through coordinated movement in all limbs.

### Spinal cord excitability after 5-weeks of training

We found no strong group effect for spinal cord excitability (assessed by H-reflex as proxy measure) to change in response to the training but 5 of 16 participants did show significant changes following the completion of the study. While the upright position is ideal for matching training conditions, group Ia presynaptic inhibition is modulated in this posture [44, 59, 60]. Since changes in reflex amplitude due to training in other work [31] shows Ia PSI as a mechanism of neuroplasticity, the choice of standing may have weakened the ability to detect change. Future work should include reflex measurement across a variety of tasks from sitting, standing, and walking [41] to better assess any changes in reflex excitability.

Amongst this population, the largest magnitudes of change were observed in individuals with PD. Following neurotrauma, there is a greater capacity for restructuring of circuitries that can be attributed to the neural plasticity in the participants [13]. The single subject analysis revealed that five (31%) of the participants showed significant changes in this study and two of the greatest changes came from the PD participants. Other research with PD presents that neurological integrity of movement is lost, therefore regaining strength and function is of utmost importance [10]. It was anticipated that those with neurological impairments would demonstrate noteworthy adaptations due to the nature of the condition and the motor systems involved with complex movements such as martial arts [9, 13, 15, 54].

### Therapeutic efficacy of a typical community martial arts training intervention

While important and useful, past martial arts training interventions provide few details about the actual content of physical performance and lacks a record of techniques used, the number of movements, and the overall effects from training with certain exposures. Here we aimed to provide insight into the quantity required. Other martial arts studies mention the style of exercises used, such as Shotokan karate with a mixture of kihon, kumite, and kata [9, 10]. A recent publication included a detailed overview of planned movements for a 10-week training, with a weekly syllabus showing the intended progression of the techniques [17] but there was no quantification of the movements done during the session. Here we add to the literature and assist future investigations by documenting specific movement volume throughout the training period.

Although the results show that our brief training program was efficacious, we still do not know the threshold stimulus for movements necessary to produce significant benefits across all measures and this should be further explored. Studies on strength training with chronic stroke participants show training-induced neural plasticity following a 5-week intervention with similar timelines to the current karate study [33, 34]. Strength training, as well as locomotor training studies in chronic stroke participants, exemplify how training load can be evaluated in a controlled setting [29, 30, 33, 34]. For example, previous work on arm cycling produced a final dosage of almost 26,000 revolutions after 5 weeks of training [30] and another study of arm and leg cycling was similar, also after 5 weeks [43]. Strength training with chronic stroke participants and neurologically intact participants showed desired results could occur in the intervention period, with dosages of approximately 375 repetitions of maximal wrist extensions [32] for improved wrist extension, 470 repetitions of maximal hand grip contractions [61] for changes in the handgrip strength, and 720 repetitions of ankle contractions [34] for changes in the legs. The calculation of training contents is simpler in controlled environment, whereas this preliminary study is the first to quantify martial arts “dosage” in this way. Additionally, balance assessments in older adults typically focus on gait stability through training with specific motor tasks [3, 54] but evaluation of specific martial arts techniques such as stance changes have not been done in the literature. Regardless, we hope that this preliminary report will contribute to a foundation for assessment of movement techniques required to produce changes in older adults with or without chronic conditions.

The martial arts training duration, frequencies, and requisite participant commitments vary within the literature, ranging from 5 weeks to 3 years [5, 9–11, 16–28]. An evaluation of over 15 martial arts training studies notably showed the differences that exist. Most of the studies had training sessions of 1 h per visit, and these occurred between 1 and 3 times a week [5, 9–11, 17–20, 23, 26, 28]. Additionally, the studies usually had around 15 participants, ranging from 11 to 23 individuals [9, 26]. For the studies that had participants training once a week, they were usually longer in duration (30 weeks–3 years) [10, 16, 22, 24]. The number of total training sessions varied from 15 to 48 sessions total [9, 11], leading to inconsistencies in the literature and preventing the understanding of necessary training exposure. The studies observed a broad range of measures such as balance, physical function, strength, and quality of life, which is congruent with the above training. What remains unknown is the minimum timing needed for efficacious community martial arts training programs.

### Limitations and recommendations

This preliminary study suggests that 5-weeks of martial arts training leads to improved movement in neurologically intact older adults and those with chronic conditions. We focused not on martial arts skills acquired through karate training but rather potentially therapeutic “side-effects” like improved balance and postural control within the “exercise is medicine” context. We are thus limited in scope about pure skill training since, although karate training was conducted, we did not directly assess skill improvements in martial arts. Instead, martial arts was the proxy for delivering training of improved balance, which could be protective against the degradation that occurs with aging.

A major limitation was the heterogeneous group and therefore strong and definitive conclusions cannot be made. This is especially the case in the participants with chronic conditions for which, due to small sample sizes, no group analysis was possible, and single subject outcomes must be interpreted with appropriate caution. Despite that, heterogeneity is also a key issue to application in the community where homogenous samples will not be found. Taken as a preliminary study, though our work adds to the exisiting knowledge and suggests further research is needed in this area. While useful data were obtained that support the use of community based martial arts training as therapeutic adjuncts in older adults, there are some important recommendations for future research:

1. Enhanced documentation and assessment of physical performance. For example, incorporating detailed enumeration of techniques as here combined with movement tracking using activity monitors (e.g. accelerometry) during training. This would be especially useful in studies assessing thresholds for changes;
2. Expanding the reach of this approach into the community to assess uptake, and implementation compared to traditional exercise alone;
3. Combining the measures here with enhanced assessment of other outcomes around mental health, such as emotional well-being and mood [9, 10];
4. The study was underpowered for some outcome measures. We suggest that future research needs to be conducted with a greater sample size with multiple intakes (something that was rendered impossible here due to the COVID 19 pandemic). Additionally, assessing groups with similar chronic conditions and determining the effective therapeutic stimulus dose for each population would be beneficial;
5. The TUG is typically used to assess individuals with chronic conditions [21] but since most participants (n = 11) were neurologically intact, there were subtle differences in the pre- and post-values. Clinical tests more specifically related to posture and balance (e.g. BERG balance test) would be better used in place of the insensitive TUG [62];
6. Assessment of dynamic balance is an important factor when the intervention trains this parameter. Future work should consider this preliminary study and related approaches to capture this effectively;
7. Simple measures of spinal cord excitability as assessed by the H-reflex were insensitive here. Suggestions are to assess multiple sizes of H-reflexes with recruitment curves, H-reflexes conditioned by somatosensory stimulation, and interlimb reflexes in future work;
8. Additional measures and assessments of fall risk should be included since recent work suggests some concerns translating physiological improvements to fall prevention, which may be related to study duration [63];
9. Measures of physiological stress (e.g. heart rate, BORG rating of perceived exertion) associated with the martial arts training itself would be helpful in future work. While there are studies addressing the physiological cost of kata training in young adults (e.g. [64]), there is little related information for older adults.

Holistic, whole-body integrated exercise programs are being implemented worldwide due to the benefits that are gained both mentally and physically. Future work should build on this and prior foundational work [9, 10] to explore the promise of mindful, enjoyable and engaging activities that contain content widely available in the community. More attempts to emphasize the psychological impacts that occur from the physiological training are necessary. We suggest participants are more likely to continue with the practices beyond our brief karate training timeline and potentially accrue additional benefits. Indeed, anecdotally, over two-thirds of the participants wanted to (and presently continue) training after the study was completed.

## Conclusions

This preliminary study demonstrates the feasibility and safety of implementing martial arts training using a community based approach. It adds to prior work showing significant changes in balance and strength suggesting that aging populations can improve function after five weeks of karate training. Further investigations of the effects of karate on spinal cord excitability and neuromuscular function are needed. Our observations contribute to the groundwork for future explorations of threshold stimulus dose, applications to neuropathology, and accessible development of martial arts interventions for older adults, leveraging programs found widely in most communities. Keeping older adults active is critical to healthy aging but, as emphasized by Pedersen and Saltin [8], “People do not move, when you tell them to. People move when the context compels them to do so. In order to enhance the physical activity level of a population, accessibility is important.” Martial arts may offer many access points to delivering useful community-level “exercise as medicine” applications across the lifespan.

## Data Availability

The datasets used and analyzed during the current study are available from the corresponding author on reasonable request.

## Abbreviations

CI: Confidence interval
H-reflex: Hoffmann reflex
PD: Parkinson’s disease
rmANOVA: Repeated measures analysis of variance
TUG: Timed up and go
tTarget: Time to target
tCenter: Time to Center
tTotal: Sum of tTarget and tCenter
